# Dysregulation of Lymphatic Endothelial VEGFR3 Signaling in Disease

**DOI:** 10.3390/cells13010068

**Published:** 2023-12-28

**Authors:** Kevin Kuonqui, Adana-Christine Campbell, Ananta Sarker, Arielle Roberts, Bracha L. Pollack, Hyeung Ju Park, Jinyeon Shin, Stav Brown, Babak J. Mehrara, Raghu P. Kataru

**Affiliations:** Plastic and Reconstructive Surgery Service, Department of Surgery, Memorial Sloan Kettering Cancer Center, New York, NY 10065, USA

**Keywords:** lymphatics, endothelial cells, lymphangiogenesis, cancer, inflammation, VEGFR-3, RTK signaling

## Abstract

Vascular endothelial growth factor (VEGF) receptor 3 (VEGFR3), a receptor tyrosine kinase encoded by the *FLT4* gene, plays a significant role in the morphogenesis and maintenance of lymphatic vessels. Under both normal and pathologic conditions, VEGF-C and VEGF-D bind VEGFR3 on the surface of lymphatic endothelial cells (LECs) and induce lymphatic proliferation, migration, and survival by activating intracellular PI3K-Akt and MAPK-ERK signaling pathways. Impaired lymphatic function and VEGFR3 signaling has been linked with a myriad of commonly encountered clinical conditions. This review provides a brief overview of intracellular VEGFR3 signaling in LECs and explores examples of dysregulated VEGFR3 signaling in various disease states, including (1) lymphedema, (2) tumor growth and metastasis, (3) obesity and metabolic syndrome, (4) organ transplant rejection, and (5) autoimmune disorders. A more complete understanding of the molecular mechanisms underlying the lymphatic pathology of each disease will allow for the development of novel strategies to treat these chronic and often debilitating illnesses.

## 1. Introduction

### VEGFR3 Signaling in Lymphatic Endothelial Cells

The lymphatic vasculature regulates immune cell trafficking and interstitial fluid homeostasis [1]. During fetal development, the proper formation of lymphatic networks is critical for the morphogenesis of nearly all organs [2]. Postnatally, stable lymphatic function is required to carry out physiologic activities of most organs, including the central nervous, cardiovascular, gastrointestinal, and integumentary systems [3]. 

**Figure 1:**
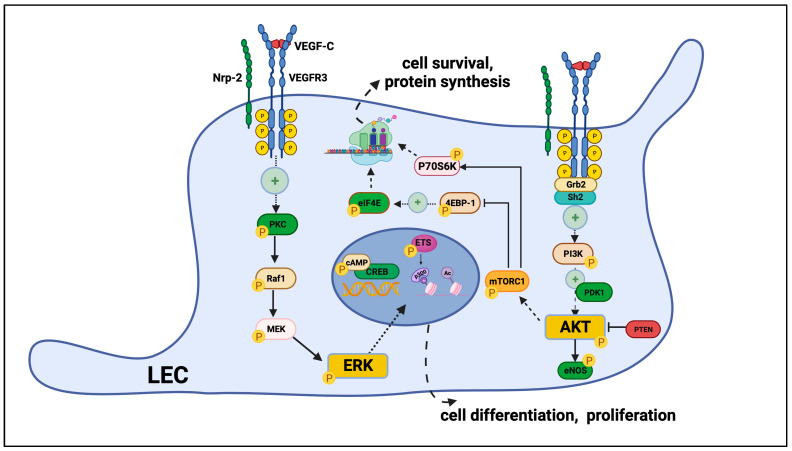
Intracellular VEGFR3 signaling in LECS in response to the VEGF-C ligand. VEGF-C-mediated activation of lymphangiogenic VEGFR3 signaling in lymphatic endothelial cells (LECs) is regulated by numerous co-receptors, adapter proteins, and secondary messengers. The PI3K-Akt and Raf-ERK pathways serve as the principal downstream effectors of activated membrane VEGFR3. Downstream Erk phosphorylation leads to the activation of nuclear transcription factors CREB and ETS to promote cellular differentiation and proliferation activities. Downstream activation of the PI3K-Akt pathways promotes the activation of ribosomal regulatory proteins elF4E and p70S6K to promote protein synthesis and cell survival functions.

Vascular endothelial growth factor (VEGF) receptor 3 (VEGFR3) is a receptor tyrosine kinase that plays a critical role in regulating growth of new lymphatic vessels (lymphangiogenesis) and new blood vessels (angiogenesis)—two processes that are essential for the development and maintenance of the vascular system. Binding of VEGF-C ligand to its cognate receptor VEGFR3 in lymphatic endothelial cells (LECs) triggers receptor homodimerization (VEGFR3-VEGFR3) or heterodimerization (VEGFR2-VEGFR3) and subsequently autophosphorylation of its cytoplasmic tyrosine kinase domains, which activate cytoplasmic secondary messengers [4,5,6]. Key VEGFR3-mediated downstream intracellular signaling interactions relevant to this review are illustrated in Figure 1. Intracellular adapter proteins including Src homology and collagen domain (Shc), growth factor receptor-bound protein 2 (Grb2), and Sh2 domain-containing protein tyrosine phosphatases play important roles in regulating downstream signaling cascade activation [7,8]. 

The Ras/mitogen-activated kinase (MAPK) and phosphoinositide-3-kinase (PI3K) signaling cascades serve as principal downstream VEGFR3 signaling effectors [9,10,11,12,13]. PI3K promotes downstream phosphorylation of protein kinase B (Akt), which in turn activates endothelial nitric oxide synthase (eNOS) and mammalian target of rapamycin (mTOR) [13,14,15]. mTOR subsequently mediates the phosphorylation of ribosomal protein S6 kinase beta 1 (p70S6K) and eukaryotic translation initiation factor 4E-binding protein 1 (4E-BP1) [16,17]. Phosphoinositide-dependent kinase 1 (PDK1) facilitates Akt phosphorylation, while phosphatase and tensin homology (PTEN) negatively regulate Akt activity [18,19]. On the other hand, protein kinase C (PKC)-dependent activation of the Raf1-MEK1/2 cascade results in downstream p42/p44 MAPK (ERK1/2) phosphorylation, which mediates the activation of the cAMP-response-element-binding protein (CREB) and Ets-domain 1 and 2 transcription factor [10,20,21,22]. Ras-GTPases serve as negative regulators of VEGF-C-mediated activation of downstream ERK signaling [23]. Classically, PI3K-Akt signaling is thought to promote the expression of pro-survival and anti-apoptotic genes, while MAPK/ERK signaling is mainly believed to mediate cellular proliferation [24,25]. Together, these pathways regulate LEC functions including proliferation, migration, and tubule formation [9,26]. 

Important molecular regulators of VEGFR3 activation include membrane coreceptors neuropilin 2 (Nrp2), which can bind either VEGF-C or class III semaphorin ligands to enhance or diminish VEGFR3 activation, respectively [27,28,29]. The EphB4 receptor facilitates VEGF-C/VEGFR3 complex internalization when stimulated by its cognate ligand EphrinB2 [30,31]. In the absence of VEGF-C, membrane integrins can transactivate VEGFR3 via recruitment of non-receptor-associated Src tyrosine kinases [32,33]. Other important modulators of VEGFR3 signal transduction include environmental mechanical forces and extracellular matrix (ECM) molecules, including fibronectin (FN), heparan sulfate (HS), and various other proteoglycans or glycoproteins [34,35,36].

In mature, healthy adults, the majority of lymphatic vessel networks throughout the body exist in a growth-quiescent state, with the exception of meningeal and intestinal lymphatics, which require continuous VEGF-C stimulation [37,38,39]. VEGFR3-mediated lymphatic remodeling can be activated as part of the inflammatory response, wound healing, obesity, tumor growth, and other physiologic or pathologic conditions [37,38,39]. Dysregulated LEC expression of VEGFR3 or its downstream mediators serves as a potential mechanism by which excessive or, more commonly, insufficient lymphangiogenesis contribute to pathological changes.

Over the past two decades, several studies have suggested that lymphatic vessel growth dysfunction contributes to numerous clinically relevant pathologies such as lymphedema; cancer; and autoimmune, metabolic, and inflammatory disorders. Therefore, understanding VEGFR3-related signaling pathways in LECs is important for developing therapeutic strategies to treat cancer, lymphedema, and other inflammatory and metabolic diseases. Targeted interventions aimed at modulating insufficient or excessive VEGFR3 signaling are actively being studied to address many of these lymphatic-associated diseases. While research in this field continues to expand, there currently is a paucity of literature outlining key pathologic findings related to dysregulated VEGFR3 signaling in lymphatic-related disorders. 

## 2. Lymphedema and Other Lymphatic Anomalies

### 2.1. Primary Lymphedema and Other Primary Lymphatic Disorders

**Figure 2:**
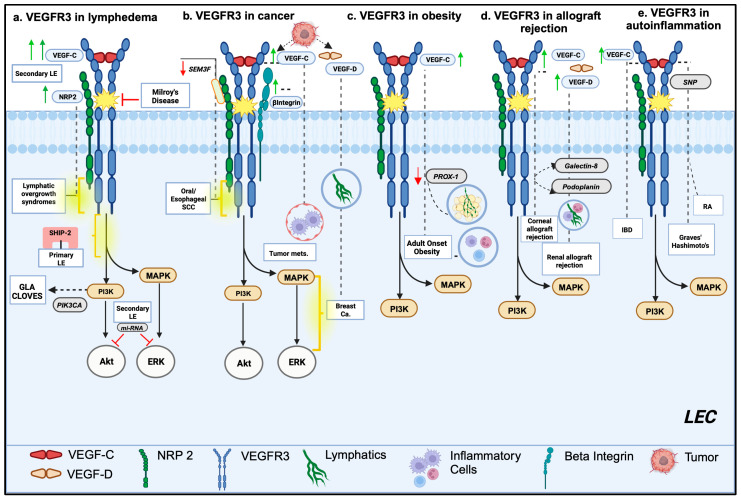
Dysregulated VEGFR3 signaling in disease states. (**a**) VEGFR3 dysregulation in lymphedema. Primary LE: defective activation of VEGFR3 is associated with Milroy’s disease. VEGFR3 and neuropilin-2 transcription is elevated in lymphovenous overgrowth malformations. Missense mutations affecting SHIP-2 protein enhance VEGF-C-induced activation of Akt and ERK in primary lymphedema. Somatic activating *PIK3CA* mutations are seen in generalized lymphatic anomaly (GLA) and congenital lipomatous overgrowth, vascular malformations, epidermal nevi, and spinal/skeletal anomalies (CLOVES) syndrome. Secondary LE: VEGF-C expression is increased in the plasma of patients with LE. Expression of micro-RNA-1236 in LECs inhibits lymphangiogenesis by downregulating VEGFR3-mediated Akt and ERK activation. (**b**) VEGFR3 dysregulation and tumor growth and metastases. Tumor-derived VEGF-C is a potent chemoattractant and enhances peritumoral lymphatic access for tumor metastasis. Downregulation of SEMA3F, a NRP2 ligand that negatively regulates VEGFR3 activation, is downregulated in esophageal SCC. Enhanced integrin β1 signaling increases peritumoral metastasis in melanoma. Tumor-derived VEGF C-D expression activates intracellular Akt and ERK signaling to promote peritumoral lymphangiogenesis in breast tumors. (**c**) VEGFR3 dysregulation in obesity and metabolic syndrome. Increased serum levels of VEGF-C are seen with metabolic disorders. Haploinsufficiency of PROX-1 is associated with excessive lymph accumulation and increased insulin and leptin levels in affected tissues. (**d**)VEGFR-3 dysregulation in transplant rejection. Inflammatory lymphangiogenesis and corneal allograft rejection is promoted by galectin-8 mediated podoplanin-VEGFR3 cross-talk. VEGF-C-D elevation promotes increased renal lymphangiogenic activity and subsequent renal transplant rejection. (**e**) VEGFR3 dysregulation in autoimmune/autoinflammatory disorders. Single-nucleotide polymorphisms (SNPs) in the gene encoding VEGF-C are identified as potential risk factors for developing Graves’ disease, Hashimoto’s thyroiditis, and rheumatoid arthritis (RA). Elevated VEGF-C and VEGFR3 mRNA expression is observed in inflammatory bowel disorders like ulcerative colitis (UC) and Crohn’s disease.

The development of the lymphatic vasculature is a well-defined process during which LEC progenitors initially bud from embryonic veins, eventually forming an early lymph sac from which all lymphatic vessels originate [40,41]. The VEGF-C/VEGFR3 signaling axis has been shown to play a critical role in the budding of early LECs from the cardinal veins [42,43,44,45]. Germline and somatic mutations have been linked to dysregulated VEGFR3 signaling in LECs, potentially leading to developmental lymphatic defects likely underlying a wide spectrum of primary lymphatic disorders (Figure 2a) [41,46]. For example, patients with Milroy’s disease, one of the most extensively studied hereditary congenital lymphedema disorders, have inactivating autosomal dominant mutations within the Fms-related tyrosine kinase 4 *(FLT4)* gene encoding VEGFR3 expression [47,48,49,50]. Some patients with Milroy-like hereditary primary lymphedema have inactivating *VEGFC* gene mutations resulting in variable degrees of lymphatic hypoplasia [51,52]. Additionally, collagen and calcium-binding EGF domain-containing protein 1 (*CCBE1*) mutations contribute to defective post-translational proteolytic activation of the VEGF-C ligand in Hennekam’s syndrome, a heritable disease characterized by congenital lymphedema and lymphangiectasia [53,54,55]. It is important to note, however, that mutations in genes encoding ADAM metallopeptidase with thrombospondin type 1 motif 3 (*ADAMTS3*) and the atypical cadherin FAT4 have also been associated with Hennekam’s syndrome, thus highlighting the complex pathophysiology underlying the development of primary lymphatic disorders [56,57]. Missense autosomal recessive *FLT4* gene mutations have also been isolated in familial lymphedema patients with dermal lymphatic hypoplasia [58]. More recently, mutations affecting other molecular signaling pathways have been increasingly implicated in the pathogenesis of several primary lymphedema disorders, which are reviewed in more detail elsewhere [59,60,61,62,63]. VEGFR3 and neuropilin-2 transcription are elevated in various lymphovenous overgrowth malformations, suggesting the underlying hyperactivation of VEGFR3 and/or its downstream signaling pathways, in contrast to primary lymphedema disorders usually characterized by attenuated VEGFR3 signaling [64]. In experimental models of cerebral cavernous malformations (CCM), deletion of programmed cell death 10 (*CCM3*), a molecular regulator of cellular apoptosis, enhances VEGFR3 expression and ERK1/2 activation in LECs, leading to hyperplastic lymphatic development [65]. Importantly, the molecular defects associated with various lymphatic overgrowth malformations usually occur intracellularly downstream from the membrane VEGFR3 activation complex, which are discussed in subsequent paragraphs. Lastly, recent studies have linked aberrant VEGFR3 function with congenital heart defect development in both humans and mice, which are described extensively in a review by Monaghan et al. [66]. For example, truncating *FLT4* gene variants have been identified in large-scale studies of patients with sporadic, non-syndromic Tetralogy of Fallot (TOF) [66,67,68,69]. Currently, there is a limited understanding of the molecular pathophysiology underlying early lymphatic disorders commonly associated with congenital heart diseases, including plastic bronchitis, chyloptysis, chylopericardium, and chylothorax [70,71]. Recently, variants of unknown significance (VUS) at the *FLT4* locus were isolated in two primary isolated congenital chylothorax patients, thus highlighting the need for further characterization of VEGFR3 signaling in these disorders [72].

Further downstream in the VEGFR3 transduction cascade, a missense germline *INPPL1* mutation affecting Sh2-domain-containing 5’-inositol phosphatase 2 (SHIP-2), an intracellular adapter protein that negatively regulates PI3K/Akt and MAPK/ERK activation, was isolated in some patients with primary lymphedema [73]. In vitro transfection of this SHIP-2 mutant enhances Akt and ERK activation following VEGF-C treatment. However, despite increased VEGFR3 signaling, mutant LECs have impaired migration, tube formation, and matrix adhesion, possibly due to increased LEC apoptosis resulting from excessive ERK activation [73].

Mutations, mostly of somatic origin, in the phosphoinositide-3-kinase (PI3K) pathway have also been identified in various lymphatic malformations. For example, somatic *PIK3CA* mutants have been reported in generalized lymphatic anomaly (GLA), a condition characterized by the development of multifocal or diffuse lymphatic lesions [74]. In a mouse model of GLA, hyperactive PI3K signaling resulted in lymphatic hyperplasia and impaired lymphatic transport [74]. In congenital lipomatous overgrowth, vascular malformations, epidermal nevi, and spinal/skeletal anomalies/scoliosis (CLOVES) syndrome, a disease affecting multiple organ systems, somatic activating PI3K mutations elicit pathologic lymphatic overgrowth anomalies [75,76]. Recently, Martinez-Corral et al. demonstrated that *PIK3CA^H1047R^* mutant-mediated microcystic lymphatic malformation overgrowth depends on intact VEGF-C/VEGFR3 and mTOR signaling in vivo, suggesting upstream signaling molecules play distinct roles in upregulating lymphangiogenic activities [77]. Somatic hyperactivating Akt and somatic inactivating PTEN mutations in Proteus syndrome also elicit similar phenotypic effects [74,78,79].

Alterations in the Ras signaling pathway may cause familial or sporadic lymphatic dysfunction, a common feature of ‘Rasopathy’ syndromes. For example, both germline and somatic inactivating mutations affecting H-Ras, N-Ras, K-Ras, and Son of sevenless homolog 1 (SOS1) have been linked to development of chylothorax and chylous ascites [75,80]. Activating somatic *KRAS* mutations have been reported in Gorham–Stout disease (GSD), where ectopic lymphatics destructively infiltrate bony structures [81]. The Dellinger group recapitulated this ectopic lymphatic growth phenotype in murine bone by generating a hyperactive *KRAS^G12D^* mutant [82]. Functionally, these mutant lymphatics exhibit decreased in vivo vessel branching, increased vessel diameter, and reduced valve formation that are hypothesized to contribute to chylothorax-related death in these mice [82]. Blockade of the downstream Ras effector MEK1/2 using trametinib ameliorated the GSD phenotype, further confirming the role of hyperactive Ras signaling in LEC dysfunction. Gain-of-function Raf mutants commonly seen in central conducting lymphatic anomalies, and Noonan syndrome, a developmental disorder caused by germline mutations in one of several Ras pathway members, lead to cutaneous lymphatic malformations and lymphangiectasia in translational animal models [75,83,84,85,86]. Additionally, loss of negative Ras regulators, such as the GTPase activator p120-RasGAP (RASA1), may result in heritable lymphatic overgrowth phenotypes [23]. These experimental findings are clinically relevant, given that altered MEK/ERK RNA expression has been detected in blood samples from lymphatic malformation patients [87].

Using whole-exome sequencing, a novel variant of the EphB4 receptor, which regulates VEGFR3 internalization, was isolated in affected family members with central conducting lymphatic anomalies across four generations [88]. In zebrafish, transfection of this EphB4 mutant results in aberrant lymphatic vessel branching and increased mTORC1 and p70-S6K phosphorylation [88]. Rather unexpectedly, mTORC1 and MEK blockade both restored lymphatic branching morphology, thus also implicating EphB4 crosstalk with MAPK signaling [88]. Previous reports of EphrinB2-EphB4-p120-RasGAP-axis-driven hyperactivation of the mTORC1 pathway further illustrate the role of non-canonical Ras/MAPK-PI3K cross-talk in the regulation of downstream VEGFR3 signaling in LECs [89]. Further characterization and identification of dysregulated VEGFR3 molecular effectors may therefore enable the development of therapies to treat primary lymphedema disorders. 

### 2.2. Secondary Lymphedema

VEGF-C, neuropilin-2, and FOXC2 polymorphisms have been identified in patients with breast cancer-related lymphedema (BCRL), suggesting that baseline differences in VEGFR3 activation or lymphatic function may contribute to the risk of developing post-surgical lymphedema [90,91]. This concept is supported by the finding that Prox-1 haploinsufficient mice with underlying subclinical lymphatic dysfunction have an increased propensity for developing lymphedema after lymphatic injury [92]. However, BCRL does not appear to be caused by a deficiency of VEGF-C because VEGF-C expression is increased in tissue biopsies and in the plasma of patients with lymphedema and in animal models of the disease [93,94,95]. Concordantly, VEGF-C overexpression in a mouse model of lymphedema results in a more rapid onset and more severe swelling due to increased vascular permeability and inflammation [96]. VEGF-C overexpression to treat BCRL led to inconclusive effects and has been largely abandoned as a therapeutic approach [97]. Genetic polymorphisms in inflammatory mediators such as IL-4, IL-10, and NF-κB confer varying degrees of risk or protection against the development of secondary lymphedema, highlighting the importance of immune responses in regulating lymphatic function [91]. Together, these findings suggest that dysregulated VEGFR3 signaling or other mechanisms impair lymphatic regeneration and function in patients who develop secondary lymphedema. 

Recent studies have suggested that alterations in regulatory microRNA (miRNA) expression may mediate the pathophysiologic features of various disease states, such as dermal inflammation, fibrosis, and immune dysregulation [98]. Expression of *miR-1236* in LECs is associated with the inhibition of lymphangiogenesis via VEGFR3, Akt, and ERK downregulation [99] (Figure 2a). Similarly, the expression of *miR128-3p* is associated with reduced LEC proliferation via the attenuation of intracellular ERK and Ca^2+^ signaling [100]. A recent study showed that the expression of *miR199a-3p* and *miR151a-3p* is increased in the serum of patients with BCRL compared to serum of breast cancer patients without BCRL or healthy controls [101]. Interestingly, the expression of these microRNAs temporally correlates with lymphedema onset and is hypothesized to increase lymphedema risk by interacting with transforming growth factor-β (TGF-β), PI3K-Akt, and MAPK signaling cascades [101]. 

In conclusion, accumulating evidence in mice and humans shows that VEGFR3 dysregulation along with increased VEGF-C ligand is observed in secondary lymphedema, possibly due to accompanying chronic inflammatory conditions. Thus, therapies controlling inflammation might hold more potential in reversing VEGFR3 dysregulation and making use of the excess lymphangiogenic ligands for lymphatic regeneration. More detailed studies are warranted to understand the paradoxical condition of excess VEGF-C in lymphedema tissues and yet poor lymphatic function. This indirectly implicates that the problem might be in the dysregulation of VEGFR3 rather than the shortage of VEGF-C.

## 3. Tumor Growth and Metastatic Environment 

Lymphangiogenesis and peritumoral lymphatics are associated with an increased risk of metastasis in several cancers including squamous cell cancer, breast cancer, and gastric cancer [102,103,104,105,106,107,108]. Tumoral lymphangiogenesis is fostered by tumor-derived VEGF-C and other lymphangiogenic growth factors such as angiopoietin 2 and fibroblast growth factor 2, which functionally interact with VEGFR3’s downstream effectors. Peritumoral lymphatics enhance access to local lymph nodes for tumor metastasis [109,110,111]. VEGF-C is also a potent chemoattractant for macrophages and other inflammatory cells that produce VEGF-C and modulate the tumor microenvironment [112,113]. Breast tumors also indirectly stimulate peritumoral lymphangiogenesis by inducing podoplanin expression in nearby macrophages by activating CLEC2A carbohydrate-binding receptors on LECs [114].

Aberrant expression of key molecular VEGFR3 regulators in malignant tumors further implicates lymphatic participation in metastasis progression (Figure 2b). In oral squamous cell carcinoma, elevated neuropilin-2 (Nrp2) expression is positively associated with tumor stage, lymphovascular invasion, and lymph node metastasis [115]. The expression of semaphorin 3F (*SEMA3F*), a Nrp2 ligand that negatively regulates VEGFR3 activation, is downregulated in esophageal squamous cell carcinoma and is associated with increased VEGF-C and Nrp2 expression [116]. Decreased semaphorin 3F expression correlates with nodal metastasis and poorer survival outcomes [116]. In hypopharyngeal cancer specimens, the elevated expression of eukaryotic translation initiation factor 4E (eIF4E), a downstream mTOR effector, is associated with increased lymphatic density within metastatic lymph nodes [117]. Enhanced integrin B1 signaling—a regulator of VEGFR3 transactivation—in peritumoral lymphatics increases the metastasis of B16 melanoma cells in mice [118].

Tumor cells also mediate lymphatic vessel recruitment by secreting various paracrine signaling molecules that act in parallel to VEGFR3. For example, tumor VEGF-D synthesis upregulates C-C motif chemokine receptor type 10 (CCR10) expression and increases LEC migration towards tumor-derived chemokine ligands 27 and 28 (CCL27/28) [119]. Similarly, tumor VEGF-C-mediated stimulation of LEC C-X-C motif chemokine receptor 4 (CXCR4) expression increases chemotactic responsiveness to peritumoral CXCL12 gradients [120]. In turn, VEGF-C-VEGFR3 signaling cascades induce LEC chemokine production, which reciprocally influences tumor activity [121,122,123]. The interaction of LEC-derived chemokine CCL21 with tumor CCR7 receptors promotes the formation of tumor–lymphatic interfaces that contribute to metastasis [102]. In in vitro studies of human skin cancers, IL-6 expression in LECs promotes tumor cell proliferation [109,124]. Breast tumor cells secrete PGE2 and induce peritumoral lymphangiogenesis in part by promoting autocrine VEGF-D expression and activating intracellular Akt and ERK signaling in murine and human LECs [114,125,126]. 

Although most studies report that lymphatic vessels primarily serve as enhancers of tumor metastasis, several studies have suggested that lymphatics may promote local antitumor effects by facilitating the presentation of tumor-associated antigens to the immune system [19]. Using a B16 melanoma model, Kimura et al. showed that mice with k-Cyclin deletion-mediated lymphatic dysfunction exhibited a decreased number of tumor-associated antigens in draining lymph nodes and increased primary tumor growth [127]. Transfer of CD8+ T cells from tumor-draining lymph nodes of *kCYC^+/−^* mice led to decreased immune cytotoxic activity against tumor cells in vitro and in vivo [127]. Despite increased primary tumor growth, there was a significant decrease in tumor metastasis, suggesting lymphatics regulate immune cell and tumor cell trafficking via distinct mechanisms [127]. B16 melanoma implantation studies in K14-VEGFR3-Ig and *Chy* mice further support these findings and show that abnormal skin lymphatics are associated with decreased intratumoral leukocyte infiltration and increased primary tumor growth [128]. Our research group has shown that the inducible ablation of peritumoral lymphatic vessels leads to increased peritumoral immunosuppressive cytokine expression, increased tumor PD-L1 expression, and decreased intratumor CD8+ T cell infiltration, which functionally correlate with increased tumor growth, suggesting tumor-associated lymphatics play active roles in regulating tumor immune responses [129]. Conversely, Song et al. found that increased VEGF-C-driven meningeal lymphatic drainage leads to improved glioblastoma tumor clearance in mice [130]. Zhou and Ma later showed that VEGF-C/VEGFR3-mediated CCL21 expression promotes dendritic cell trafficking and subsequent CD8+ T cell activation, leading to attenuated tumor growth in mouse glioma models [131,132]. A recently developed vaccine that overexpresses VEGF-C induces lymphangiogenesis and enhances T-cell-mediated antitumor immunity and sustained attenuated tumor growth in B16 melanoma mouse models [133]. Of note, some studies have reported that elevated lymphatic vessel density mediates increased immune tolerance to tumors [134,135]. 

Despite the evidence supporting the two faces of tumor lymphatic vessels, i.e., promoting tumor growth and progression and accelerating anti-tumor immune responses, it is not clear what warrants this dual nature. It will be interesting to understand whether tumor type dictates the pro or anti-tumor lymphatic function. Further research is needed to understand the dual nature of lymphatic vessels in regulating tumor growth and progression. Independent of the pro- or anti-tumor nature of tumor lymphatic vessels, it is getting clear that the dysregulation of VEGFR3 is critical in tumor lymphatics, and targeting VEGFR3 to regulate tumor lymphatic vessels could be an ideal way going forward. 

## 4. Obesity and Metabolic Syndrome 

The negative effects of obesity on lymphatic function have been extensively reported, but fewer studies have focused on characterizing the reciprocal contribution of dysregulated lymphangiogenic activity to the development of obesity and metabolic syndrome [136,137,138,139,140]. A link between lymphatic vessel dysfunction and metabolic syndrome is suggested by case series reporting increased serum levels of VEGF-C in patients with metabolic disorders [141,142,143]. Recently, the Rockson group published a single-center retrospective cohort analysis identifying a positive association between the presence of clinically diagnosed lymphatic disorders (lipedema, secondary lymphedema, or lymphovascular disease) and diabetes, further strengthening the hypotheses of lymphatic involvement in metabolic disease pathogenesis [144]. 

One of the earliest studies to implicate lymphatic dysfunction in the pathogenesis of adult-onset obesity was the discovery that haploinsufficiency of the multisystem developmental regulator Prox-1 causes this phenotype, along with the increased incidence of hepatic steatosis and elevations in circulating insulin and leptin [145] (Figure 2c). Other studies showed that VEGFR3 haploinsufficent mice (i.e., *Chy* mice) also have increased subcutaneous fat deposition [146,147]. Harvey et al. proposed that excessive lymph accumulation in affected tissues enhances lipid storage in existing adipocytes and increases adipogenesis, ultimately leading to increased ectopic fat deposition [145]. The same research group later validated this interpretation by demonstrating that the restoration of Prox-1 activity rescues this adult obesity phenotype in mice [148]. Consistent with these studies, our group showed that lymphatic fluid stasis resulting from surgical lymphatic disruption in mouse tail skin promotes increased adipose differentiation marker expression in affected tissues, further implicating lymphatic fluid stasis in adipogenesis [149]. These experimental studies collectively suggest that insufficient adipose tissue lymphangiogenesis or impaired lymphatic function may contribute to increased fat accumulation. In agreement with the above, Chakraborty et al. showed that adipose-specific transgenic VEGF-D overexpression in mice fed a high-fat diet (HFD) increases lymphangiogenesis, decreases macrophage infiltration, and improves systemic glucose and lipid metabolism markers [150]. 

Lymphatic VEGFR3 signaling is also a critical regulator of lipid and cholesterol transport in multiple cell types. The genetic deletion of VEGF-C results in lymphatic vessel regression within the lacteals and intestinal wall and decreases systemic triglyceride and cholesterol levels [151]. Using *Chy* mice and anti-VEGFR3 monoclonal antibodies, Martel et al. demonstrated the role of lymphatic VEGFR3 signaling in regulating macrophage reverse cholesterol transport from peripheral tissues [152]. In subsequent studies, the same researchers found that reversing hypercholesterolemia-induced lymphatic transport dysfunction with exogenous VEGF-C decreases the progression of atherosclerotic plaques in atherogenic mice on an HFD [153]. Deletion of the fatty acid transporter CD36 in LECs results in attenuated VEGF-C-induced Akt activation, impaired cellular oxidative metabolism, junctional VE-cadherin destabilization development of leaky gut lymphatics, and obesity in mice, suggesting that intracellular VEGFR3 signaling may be an attractive target for translational studies [154]. 

Obesity affects lymphatic function by promoting a chronic low-level inflammatory state in perivascular lymphatic tissues [136,155,156]. While studying the relationship between mesenteric lymphatic dysfunction and metabolic syndrome, Cao et al. reported that both HFD-fed obese mice and humans with obesity had leaky mesenteric lymphatics, along with elevated VEGF-C expression within extravasated lymph and neighboring visceral adipose tissue [157]. An intestinal lymphatic-targeted COX-2 inhibitor (orally administered) reduced mesenteric VEGF-C expression and concomitantly restored mesenteric lymphatic function via attenuation of macrophage-derived growth factor secretion, highlighting the importance of immune cells in regulating gastrointestinal VEGF-C homeostasis [157]. Transgenic overexpression of VEGF-C driven by the epithelial keratin-14 promoter in mouse dermal tissues results in increased weight gain and the development of insulin resistance in mutant mice, due in part to increased infiltration of pro-inflammatory M1 macrophages within subcutaneous white adipose tissue [158]. It will be interesting to observe how VEGFR3 expression and signaling on LECs change in the state of chronic low-grade inflammation with abundant VEGF-C in the tissues. 

Obesity-associated inflammation also reduces lymphangiogenesis. Analyzing sorted LECs from HFD-fed C57BL/6J obesity-prone mice showed that the expression of VEGFR3 is decreased in obesity, while HFD-fed BALB/cJ and myostatin null (*MSTN^ln^*) obesity-resistant mice had no significant changes to their lymphatics [159,160,161]. Based on findings of increased peri-lymphatic lipid droplet accumulation in HFD obese mice, we performed in vitro studies to determine the effects of long-chain free fatty acids on lymphatic function. LECs treated with stearic acid exhibited decreased VEGFR3 expression and downstream Akt and eNOS activation, which was reversible using pharmacologic PTEN blockade, illustrating a mechanism by which lymphangiogenesis can be modulated in disease states without the additional recruitment of immune cells [19,159]. These studies suggest obesity-associated inflammation confers increased LEC resistance to VEGFR3 activation, further contributing to the progression of metabolic dysfunction. 

In conclusion, research shows that obesity decreases lymphatic function by the downregulation of VEGFR3 signaling via chronic low-grade inflammation, and, conversely, decreased lymphatic function also contributes to lipid accumulation causing obesity. Thus, specific targeting of VEGFR3 signaling to improve lymphatic function either by ligand (VEGF-C/D) overexpression or by controlling chronic inflammation with lymphatic dysfunction related obesity and additional metabolic disorders. 

## 5. Transplant Allograft Rejection 

The lymphatic vasculature is thought to contribute to chronic organ transplant rejection by promoting antigen-presenting cell (APC) trafficking and activation of adaptive immune responses (Figure 2d). This pathophysiologic process has been extensively studied in the mammalian cornea because it is avascular and immune-privileged under healthy conditions but becomes vascularized and inflamed under pathologic conditions [162,163,164,165,166,167]. In a murine allogeneic corneal transplant model, Dietrich and colleagues found that lymphangiogenesis had a greater effect on immune allograft rejection rates than angiogenesis [167]. Inhibition of angiogenesis using VEGF-Trap resulted in a lesser degree of graft survival improvement than the inhibition of lymphangiogenesis using either of the two VEGFR3 inhibitors [167]. Interestingly, galectin-8, a carbohydrate-binding protein found in LECs, has also been identified as a promoter of corneal allograft rejection via the upregulation of inflammatory lymphangiogenesis by mediating podoplanin-VEGFR3-integrin crosstalk [168].

Similarly, within a renal allograft mouse model, spontaneous regeneration of the renal lymphatic vasculature after surgery has been linked to an increase in the systemic trafficking of CCR7+ APCs involved in adaptive immune responses and progression of chronic transplant rejection [169]. Upon analyzing biopsies of renal transplant patients, these same researchers found patients experiencing chronic transplant rejection displayed greater lymphatic vessel area but not vessel number in their kidneys compared to transplant patients who were not experiencing organ rejection [169]. Additionally, VEGF-C, VEGF-D, and fibroblast growth factor 2 (FGF-2) expression was elevated within these transplant rejection patients, reflecting increased renal lymphangiogenic activity [169]. In another mouse renal transplant study, downstream inhibition of the VEGFR3 effector mTORC1 using sirolimus reduced lymphangiogenic activity, which elicited greater attenuation of chronic allograft transplant injury responses compared to calcineurin inhibition, thus implicating intracellular VEGFR3 signaling in the pathophysiologic development of chronic kidney rejection [170]. 

In cardiac allograft transplantation, lymphangiogenesis similarly supports immune cell infiltration to facilitate rejection. In a murine model, post-allograft ischemia–reperfusion injury stimulated VEGFR3 expression in LECs and promoted increased CD4+ T cell, CD8+ T cell, and ED1+ macrophage, and myeloperoxidase (MPO)+ neutrophil infiltration [171]. Accordingly, and in line with the protective effects of blocking VEGFR3 signaling in other transplant settings, inducible LEC-specific deletion of VEGFR3 prior to transplant surgery improves graft survival rates [171]. In another study, increased lymph flow and enhanced migration of donor passenger leukocytes from cardiac allografts to recipient draining lymph nodes was associated with increased allograft CD8+ T cell infiltration [172]. Furthermore, cardiac allograft vasculopathy, determined by the severity of arterial luminal occlusion, correlated with CD8+ T cell infiltration density and lymphatic vessel area, suggesting lymphangiogenic activity contributes to graft loss [172]. Inhibition of LEC VEGFR3 signaling in a rat cardiac allograft model reduced the tissue expression of CCL21, a chemokine promoter of dendritic cell migration, which reduced inflammatory cell infiltration and prolonged graft viability [123]. VEGFR3 has also been shown to promote the migration of peripherally injected naïve CD4+ T cells to draining lymph nodes by regulating extracellular heparan sulfate expression and resultant CCL21 gradients in a PI3K-dependent manner [173]. 

Many transplant rejection models conceptualize lymphangiogenesis as a pathogenic driver of allograft failure. However, based on experimental findings of decreased pulmonary lymphatic density and increased hyaluronan (HA) fragment accumulation after mouse lung transplantation, Cui et al. hypothesized that impaired hyaluronan clearance from inadequate lymphatic drainage may mediate acute lung transplant rejection [174]. Compared to controls and isografts, lung allografts exhibited a significantly higher proportion of apoptotic LECs and lower nuclear proliferation marker expression, thus confirming impaired lymphangiogenic capacity [174]. In contrast to other transplant settings, daily intravenous VEGF-C156S (the lymphangiogenesis-specific form of VEGF-C) injections enhanced lymphatic drainage and HA clearance and attenuated acute rejection responses in treated mice [174]. These findings appear to be clinically relevant, since reductions in tissue HA levels following treatment for acute lung transplant rejection were associated with improved respiratory functional outcomes [174]. 

In conclusion, corneal, renal, and cardiac transplant rejections are mainly due to activated VEGFR3 signaling, and blocking VEGFR3 signaling is a potential therapeutic target for longer transplant survival. On the contrary, lung transplant survival is proper lymphatic drainage dependent, and thus activation of VEGFR3 signaling through pro-lymphangiogenic agents seems to be critical. Further research has yet to reveal how lung transplants differ from other organ transplants in terms of opposite lymphatic and VEGFR3 signaling requirements. 

## 6. Autoimmune and Autoinflammatory Disorders 

Altered VEGFR3 signaling and the resulting lymphatic vessel dysfunction have also been implicated in the pathogenesis of multiple autoinflammatory disorders, including several glandular autoinflammatory conditions. Single-nucleotide polymorphisms (SNPs) in the gene encoding VEGF-C have been identified as potential risk factors for developing autoimmune thyroid diseases, including Grave’s disease and Hashimoto’s thyroiditis [175]. Within the minor salivary glands of primary Sjogren’s syndrome patients, newly expanded lymphatic capillaries display increased VEGFR3 expression and increased periductal inflammatory cell infiltration [176] (Figure 2e). Behçet’s disease patients experiencing uveitis exhibit higher circulating levels of soluble VEGFR3 (sVEGFR3) and a lower ratio of VEGF-C/sVEGFR3, suggesting dysregulated lymphangiogenesis [177].

Research shows that systemic lupus erythematosus (SLE) and systemic sclerosis (SS) display cutaneous lymphatic dysfunction with limited available information on VEGFR3 expression. SLE patients exhibit dilated cutaneous lymphatic vessels without significant changes in lymphatic density [178]. Ambler et al. recapitulated these findings in an ultra-violet radiation (UVR)-sensitive mouse model in which SLE is induced via chronic epicutaneous imiquimod treatment [178]. LEC-specific *PTEN* gene knockout, which enhances intracellular VEGFR3 activation and resulting lymphatic drainage, in imiquimod-treated mice following UVR exposure led to decreased inflammation and B-cell responses in draining lymph nodes [178]. Cutaneous biopsies from systemic sclerosis (SS) patients with multi-organ fibrosis display diminished lymphatic vessel density but increased VEGFR3 and VEGF-D mRNA expression compared to healthy controls [179]. Another study in SS patients noted an inverse correlation between the number of fingertip ulcers and cutaneous biopsy lymphatic vessel counts [180]. In vitro treatment of wildtype LECs with SS-patient-derived serum resulted in reduced VEGFR3 expression and impaired migratory, proliferative, and tube-forming capacity [181].

While lymphangiogenesis is usually regulated by VEGF-C/VEGFR3, a VEGF-A overexpression mouse model of psoriasis displayed persistently increased lymphatic vessel size, tortuosity, and proliferation as part of delayed-type hypersensitivity reactions. These changes were reversible with combined VEGFR1-VEGFR2 blockade, thereby implicating excessive LEC VEGFR2 signaling in the pathogenesis of at least one type of inflammatory skin disease [182]. 

Within a mouse model of alopecia areata, an autoimmune condition, lymphatic vessels within affected dermal tissues were distended, suggesting lymphatic contribution to hair growth [183]. Transgenic overexpression of VEGF-C and resulting increased dermal lymphatic density promoted prolonged anagen hair follicle growth via paracrine activation of dermal papilla cells [184,185]. Conversely, the expression of soluble VEGFR3 (sVEGFR3) extracellular domains produced the opposite results, indicating that VEGFR3 underactivity limits hair growth [184]. Additionally, recent seminal finding related to hair follicle stem cells (even though not directly related to alopecia) indicate that hair follicle stem cell (HFSC)-associated lymphatics have also been shown to promote stem cell quiescence [186]. Diphtheria-toxin-mediated dermal lymphatic ablation promoted precocious telogen-to-anagen transitions in hair follicles, which was also recapitulated following soluble VEGFR3 antibody treatment, implicating VEGFR3 signaling in this process [186]. In turn, HFSCs reciprocally regulate lymphatic activity via angiopoietin-like protein 7 and angiopoietin-like protein 4 expression [186]. Further studies in this direction are needed to fully characterize the role of lymphatic vessels in hair growth and regeneration and the link to autoimmunity in this context. 

Certain VEGF-C SNPs confer the increased risk for developing rheumatoid arthritis (RA), suggesting a potential contribution of dysregulated VEGFR3 signaling [187]. Additionally, TNF-α, the primary driver of RA progression, has been shown to increase VEGF-C expression in the affected joint synovial fluid of RA patients, potentially disrupting endogenous growth factor gradients [188]. Several experimental RA models propose that lymphatic insufficiency may worsen disease progression, which is supported by clinical findings of impaired lymphatic clearance of indocyanine green (ICG) dye within the affected hands of RA patients and experimental findings of decreased lymphatic vessel maturity within the inflamed joints of transgenic TNF-expressing mice [189,190]. Within translational models of inflammatory arthritis, VEGFR3 blockade results in decreased lymphangiogenesis, impaired afferent lymphatic drainage, and increased synovial volumes in transgenic tumor necrosis factor (TNF)-overexpressing mice [191]. Conversely, treatment with adenovirus expressing VEGF-C has been shown to increase intraarticular lymphatic vessel density and concomitantly reduce joint swelling and inflammation [192]. Together, these findings indicate that impaired lymphangiogenesis and lymphatic function promotes the pathogenesis of RA [191,192]. In contrast, in patients with adult-onset Still’s disease, an incompletely understood autoinflammatory disorder characterized by fever, arthralgias, and lymphadenopathy, circulating VEGF-C has recently been found to be elevated [193]. Interestingly, serum VEGF-C concentrations correlated with active symptoms, inflammatory cytokine levels, and disease severity markers [193].

Elevated VEGF-C, VEGFR3, podoplanin, and LYVE-1 mRNA expression has been observed within the inflamed colonic mucosa of ulcerative colitis (UC) and Crohn’s disease (CD) patients [194,195]. This has been reproduced in dextran sodium sulfate (DSS)-induced mouse models of inflammatory bowel disease (IBD), in which the intestinal lymphatics exhibit increased vessel density, elevated VEGFR3 expression, and vessel dilation, suggesting that overactive lymphangiogenesis may be involved in disease pathogenesis [196,197]. However, this hypothesis is complicated by the finding that VEGFR3 blockade induces the development of dilated and tortuous lymphatics in the IL-10 knockout model of spontaneous inflammatory bowel disease (IBD), which was shown to worsen intestinal inflammation [198]. To better understand these relationships, several research groups have induced VEGF-C overexpression, leading to improvement in some studies and worse intestinal inflammation in others [196,199]. Indeed, it remains unknown if these histopathologic findings are causative or compensatory in nature, making it difficult to determine if insufficient versus excessive lymphangiogenesis is to blame [200]. However, dysfunctional lymphatics clearly exert some influence on intestinal inflammatory disease progression. 

In conclusion, autoimmune pathologies seem to be highly heterogeneous, showing a disease specific up- and downregulation of VEGFR3 signaling. While SLE, SS, and alopecia exhibit reduced VEGFR3 signaling, RA, UC, CD, and IBD display increased VEGFR3 signaling; thus, both pro- and anti-lymphatic therapies are in play, depending on the disease type. For future studies, it will be interesting to elucidate the basis of the heterogeneity in VEGFR3 signaling between different autoimmune conditions to specifically target lymphatic vessels and VEGFR3 signaling.

## 7. Conclusions

Aberrant VEGFR3 signal transduction and altered lymphangiogenesis or lymphatic function may contribute to the development of many diseases, as summarized in Table 1. This effect is likely due to the central role of the lymphatic system in tissue fluid drainage, fat absorption, and immune cell trafficking, as well as the propensity of lymphatic vessels to become injured by chronic inflammatory reactions. The heterogeneity in VEGFR3 signaling across different pathologies shows that not all diseases arise uniformly with respect to alterations in lymphatic growth and function. For example, diseases like secondary lymphedema and obesity largely show decreased VEGFR3 signaling despite increased VEGF-C ligand expression in affected tissues, indicating a probable development of VEGF-C resistance in these pathologies, akin to the phenomenon of insulin resistance [201]. Thus, therapeutic interventions in these diseases should be focused on enhancing VEGFR3 signaling by regulating chronic inflammation rather than exogenous lymphangiogenic ligand supply. Demonstrating the concept of VEGF-C resistance in lymphatic pathologies and understanding the molecular basis of such phenomena could be a potential new direction for future studies. In contrast to lymphedema and obesity, many cancers and organ transplant rejection cases, when viewed from a lymphatic perspective, arise mainly due to increased VEGFR3 signaling and the pathologic activation of lymphatic growth. In these conditions, therapeutic strategies should rely on dampening VEGFR3 signaling. Finally, autoimmune pathologies involving lymphatic dysfunction appear to be a mixed bag, with some pathologies showing increased while others show decreased VEGFR3 signaling, therefore necessitating disease-specific therapeutic interventions. Overall, understanding the cellular mechanisms that regulate the crosstalk between lymphatic vessels and other tissues is an important research goal. This understanding may lead to novel targeted therapies that can impact not only primarily lymphatic diseases such as lymphedema but also a host of common diseases including obesity, metabolic syndrome, tumors, and autoimmune disorders.

## Figures and Tables

**Table 1:** Proposed VEGFR3-signaling-related molecular alterations in lymphatic-related diseases.

Disease	Clinical Manifestations	Histopathologic Findings	Proposed VEGFR3-Related Molecular Pathology	Selected References
Milroy/Milroy-like primary lymphedema	Lower extremity swelling	Hypoplastic lymphatics	VEGF-C, VEGFR3, INPPL1 gene mutations	[48,49,51,52,58,73,202]
Hennekam’s syndrome	Generalized lymphedema and lymphangiectasia, variable intellectual disability, characteristic facial dysmorphic features	Lymphatic vessel dysplasia	CCBE-1, ADAMTS3 gene mutations	[54,55,56]
Isolated lymphovenous malformations	Congenital lymphatic malformations, cerebral cavernous malformations (CCM)	Hyperplastic lymphatics	Increased cellular VEGFR3, neuropilin-2, ERK 1/2 activity	[64,65]
Generalized lymphatic anomaly (GLA)	Diffuse or multi-focal lymphatic malformations; cutaneous/soft tissue edema; chylous thoracic effusions, ascites, lymphorrhea	Hyperproliferative, dilated lymphatics	PIK3CA mutation (hyperactivating)	[74]
CLOVES syndrome	Capillary, venous, lymphatic vascular malformations; thoracic lipomatous hyperplasia; asymmetric limb growth	Macrocystic and microcystic malformations, or combined lymphatic lesions w/disorganized channels	PIK3CA mutation (hyperactivating)	[76,203]
Proteus syndrome	Cutaneous, Musculoskeletal, and vascular tissue overgrowth lesions	Hyperplastic lymphatics, abnormal lymphovascular channels	AKT1 mutation (hyperactivating) PTEN (inactivating)	[78,79]
Gorham–Stout disease	Spontaneous, progressive osteolysis; soft tissue lymphangioma; chylothorax	Proliferative ectopic lymphatics within bony structures	KRAS mutation (activating)	[81,82]
Noonan syndrome	Lymphedema of bilateral lower limb and genitals; posterior cervical hygroma	Dilated hyperplastic lymphatics	Ras-Raf, PTPN11, SHP2 mutations	[84,85,86]
Central conducting lymphatic anomaly (CCLA)	Abnormal lymphatic drainage by large vessels within the trunk	Dilated channels, central lymphatic obstruction	ARAF, EPHB4 mutations	[83,88,204]
Tumor Metastasis	Clinical Manifestations	Histopathologic Findings	Proposed VEGFR3-Related Molecular Pathology	Selected References
Breast cancer	Local tissue infiltration and metastasis to distant organs	Peri-tumoral lymphatic growth and tumor invasion of neighboring lymph nodes	PGE2-EP4 axis stimulation, CLEC2A activation, VEGF-C overexpression	[114,125,126]
Melanoma	Local tissue infiltration and metastasis to distant organs	Aberrant distribution of melanocytes, Pagetoid spread, dyscohesive nests of melanocytes	ARF6 upregulation, integrin B1 upregulation, k-Cyclin deletion	[118,127,128,133,134,135]
Oral SCC	Local tissue infiltration and metastasis to distant organs	Tumor budding, perineural and vascular invasion, sarcolemma spread, tumor-infiltrating T-lymphocytes, CD68+-tumor-associated macrophages, tumor-associated tissue eosinophilia, cellular cannibalism	Neuropilin-2 upregulation, SEMA3F downregulation, VEGF-C overexpression	[108,115]
Hypopharyngeal cancer	Local tissue infiltration and metastasis to distant organs	Squamous cell (most common), submucosal tumor extension	EIF4E activation	[117]
Esophageal SCC	Local tissue infiltration and metastasis to distant organs	Keratin pearls, individual cell keratinization, intercellular bridges	SEMA3F downregulation	[116]
Cholangiocarcinoma	Local tissue infiltration and metastasis to distant organs	Abundant fibrous desmoplastic stroma	CXCR2-CXCL5 axis activation	[205]
Autoimmune Disorders	Clinical Manifestations	Histopathologic Findings	Proposed VEGFR3-Related Molecular Pathology	Selected References
Autoimmune thyroid disease	Grave’s disease; Hashimoto’s thyroiditis; dysregulated thyroid hormone secretion	Increased lymphatic vessel density	VEGF-C polymorphisms	[175,206]
Sjogren’s syndrome	Diminished lacrimal and salivary gland function, xerostomia, keratoconjunctivitis sicca, parotid gland enlargement	Expansion of lymphatic capillaries, periductal inflammatory cell infiltration	VEGFR3, VEGF-C polymorphisms	[176]
Behcet’s disease	Mucocutaneous lesions, recurrent genital ulcerations, uveitis, skin lesions	Expansion of lymphatic capillaries, periductal inflammatory cell infiltration	Increased circulating sVEGFR3, dysregulated VEGF-C/ VEGFR3 ratio	[177]
Alopecia areata	Discrete, round patches of hair loss	Dilated dermal lymphatics	Elevated vascular endothelial growth factor expression	[183]
Systemic lupus erythematosis (SLE)	Progressive multi-organ tissue fibrosis, vascular damage and inflammation	SLE: Dilated cutaneous lymphatic vessels without changes in lymphatic density	Increased VEGF-C, VEGF-D, VEGFR2, VEGFR3 activity	[178]
Psoriasis	Well circumscribed, circular, erythematous papules and plaques with dry scaling	Perivascular and dermal inflammatory cell infiltration, vascular dilatation, edema of dermal papillae, parakeratosis	Increased VEGF-A expression, VEGFR2 activation	[182]
Rheumatoid arthritis	Systemic polyarthritis, articular destruction	Chronic perilymphatic inflammation, lymphatic leakiness	VEGF-C polymorphisms, impaired VEGF-C/VEGFR3 signaling	[187,188,189,190]
Still’s disease	Autoinflammatory fever, arthralgias, lymphadenopathy, joint pain, persistent papules and plaques	Upper keratinocyte dyskeratosis, scattered superficial dermal neutrophils, vacuolar interface changes, apoptotic keratinocytes	Increased VEGF-C expression	[193]
Inflammatory bowel disease	Crohn’s disease, ulcerative colitis	Increased lymphatic vessel density	Impaired VEGFC/VEGFR3 axis signaling	[194,195,196,197,198,199,200]
Allograft Transplant Rejection	Clinical Manifestations	Histopathologic Findings	Proposed VEGFR3 Related Molecular Pathology	Selected References
Corneal transplant rejection	Progressive end-organ dysfunction, corneal edema, anterior chamber inflammation, increased intraocular pressure	Increased pathologic lymphangiogenesis	Increased VEGFR3 activation; galectin-8-mediated integrin- podoplanin-VEGFR3 crosstalk	[162,163,166,167,168]
Renal allograft rejection	Malaise, fever, oliguria, graft pain or tenderness, progressive end-organ dysfunction	Endothelial cell swelling and inflammation, dilated peri-tubular capillaries, thrombotic microangiopathy, subendothelial widening (acute), tubular hypertrophy, interstitial fibrosis, mononuclear inflammatory cell infiltrate (chronic)	Increased VEGFR3, VEGF-D, mTORC1 activation	[169,170]
Cardiac allograft rejection	Arterial luminal occlusion, diminished cardiac output, hypotension, mechanical abnormalities	CD4+ T cell, CD8+ T cell, ED1+ macrophage, myeloperoxidase +, neutrophil infiltration, perivascular infiltration, interstitial inflammatory cells Decreased pulmonary lymphatic vessel density, increased hyluronan, fragment accumulation, lymphocytic infiltrate	Increased VEGF-C/VEGFR3 activation	[123,171,172]
Pulmonary allograft rejection	Dyspnea, cough, sputum production, respiratory distress (acute)		Insufficient VEGF-C/VEGFR3 activation	[174]
Obesity/metabolic syndrome	Hypertension, hyperglycemia, visceral adiposity, dyslipidemia	Leaky lymphatics, impaired lymph drainage, lymphatic fluid stasis	Prox-1 mutations, increased adipose tissue and serum VEGF-C levels, reduced VEGFR3-AKT-eNOS activation, impaired LEC CD36 activity	[141,142,143,144,150,154,157,158]
Secondary/Acquired Lymphedema	Clinical Manifestations	Histopathologic Findings	Proposed VEGFR3-Related Molecular Pathology	Selected References
Breast-cancer-related lymphedema (BCRL)	Subcutaneous lymphatic fluid stasis and accumulation, fibroadipose skin deposition, dermal thickening	CD4+ T cell infiltration, fibrosis, immature hyperplastic, leaky lymphatics	Prox-1, VEGF-C, Neuropilin-2 mutations, elevated tissue and systemic VEGF-C expression, SHIP2 mutation, epigenetic microRNA alterations	[90,91,92,93,94,96,97,98,207]

## Data Availability

Data sharing is not applicable to this article.
